# The role of neuroimaging in the determination of brain death

**DOI:** 10.1590/0100-3984.2022.0016

**Published:** 2022

**Authors:** Diogo Goulart Corrêa, Simone Rachid de Souza, Paulo Glukhas Cassar Nunes, Antonio Carlos Coutinho Jr., Luiz Celso Hygino da Cruz Jr.

**Affiliations:** 1 Department of Radiology, Clínica de Diagnóstico por Imagem (CDPI)/Dasa, Rio de Janeiro, RJ, Brazil.; 2 Department of Radiology, Universidade Federal Fluminense (UFF), Niterói, RJ, Brazil.; 3 Department of Pathology, Universidade Federal do Rio de Janeiro (UFRJ), Rio de Janeiro, RJ, Brazil.; 4 Department of Radiology, Fátima Digittal, Casa de Saúde Nossa Senhora de Fátima, Nova Iguaçu, RJ, Brazil.

**Keywords:** Brain death, Tomography, X-ray computed, Computed tomography angiography, Magnetic resonance imaging, Magnetic resonance angiography, Morte encefálica, Tomografia computadorizada, Angiografia por tomografia computadorizada, Ressonância magnética, Angiografia por ressonância magnética

## Abstract

Brain death is the irreversible cessation of all brain function. Although protocols for its determination vary among countries, the concept of brain death is widely accepted, despite ethical and religious issues. The pathophysiology of brain death is related to hypoxia and ischemia in the setting of extensive brain injury. It is also related to the effects of brain edema, which increases intracranial pressure, leading to cerebral circulatory arrest. Although the diagnosis of brain death is based on clinical parameters, the use of neuroimaging to demonstrate diffuse brain injury as the cause of coma prior to definitive clinical examination is a prerequisite. Brain computed tomography (CT) and magnetic resonance imaging (MRI) demonstrate diffuse edema, as well as ventricular and sulcal effacement, together with brain herniation. Angiography (by CT or MRI) demonstrates the absence of intracranial arterial and venous flow. In some countries, electroencephalography, cerebral digital subtraction angiography, transcranial Doppler ultrasound, or scintigraphy/single-photon emission CT are currently used for the definitive diagnosis of brain death. Although the definition of brain death relies on clinical features, radiologists could play an important role in the early recognition of global hypoxic–ischemic injury and the absence of cerebral vascular perfusion.

## Abstract (pt)

A morte encefálica é a cessação irreversível de todas as funções cerebrais. Embora os protocolos para sua determinação variem entre os países, o conceito de morte encefálica é amplamente aceito, apesar de questões éticas e religiosas. A fisiopatologia da morte encefálica está relacionada a hipóxia e isquemia no cenário de uma lesão cerebral difusa. Também está relacionada aos efeitos do edema cerebral, que aumenta a pressão intracraniana, levando à parada da circulação cerebral. Embora o diagnóstico de morte encefálica seja baseado em parâmetros clínicos, o uso de neuroimagem para demonstrar lesão cerebral difusa como causa do coma antes do exame clínico definitivo é um pré-requisito. A tomografia computadorizada (TC) e a ressonância magnética (RM) de crânio demonstram edema difuso e apagamento de ventrículos e sulcos, associados a herniações transcompartimentais. A angio-TC e a angio-RM demonstram a ausência de fluxo arterial e venoso intracraniano. Em alguns países, a eletroencefalografia, a angiografia por subtração digital cerebral, a ultrassonografia transcraniana com Doppler ou a cintilografia/TC por emissão de fóton único são atualmente usadas para o diagnóstico definitivo de morte encefálica. Embora a definição de morte encefálica dependa de características clínicas, os radiologistas podem desempenhar papel importante no reconhecimento precoce da lesão hipóxico-isquêmica global e da ausência de perfusão vascular cerebral.

## INTRODUCTION

Brain death is defined as the irreversible cessation of all brain function, including cortical and brainstem activities^(1)^. It was first described after the advent of mechanical ventilation and cardiopulmonary support, which enabled the replacement of lost heart and lung function. The ability to artificially maintain vital body functions after the brain has irreversibly ceased to function led to a reexamination of the criteria for death^(2)^.

Despite ethical and religious issues, the concept of brain death is now widely accepted, conceptually and legally, although protocols for its definition vary among countries^(3)^ and well-defined protocols are lacking in some countries^(4)^. In some countries with such protocols, clinical examination is the primary means of determining the occurrence of brain death, ancillary tests being used only in cases of diagnostic concerns, whereas ancillary testing is mandatory in some other countries^(3)^. In Brazil, according to Resolution 2.173 of the Federal Council of Medicine, the determination of brain death requires two clinical examinations, performed by two properly trained physicians, that confirm deep unresponsive coma, the absence of brainstem reflexes (pupillary light reflex, corneal reflex, oculocephalic reflex, oculovestibular reflex, and cough reflex), with a time interval defined by patient age, as well as a positive apnea test and at least one positive ancillary test^(5)^.

The ancillary tests used to support the occurrence of brain death also vary among countries. For example, in Brazil and the United States, the approved ancillary testing methods are electroencephalography, cerebral digital subtraction angiography (DSA), transcranial Doppler ultrasound, and cerebral single-photon emission computed tomography (SPECT)—Table 1; computed tomography (CT) and magnetic resonance imaging (MRI), including angiography performed with both methods (CTA and MRA, respectively), have not yet been approved^(4,5)^. Countries such as the Netherlands and France have approved the use of CTA as an ancillary test for the determination of brain death^(6)^.

**Table 1:** Ancillary tests recommended in Brazilian Federal Council of Medicine Resolution 2.173.*

Ancillary test	Purpose
Cerebral DSA	To demonstrate the absence of intracranial flow in the internal carotid and vertebral arteries, above the ophthalmic artery and basilar artery, respectively, according to the technical standards of the Brazilian College of Radiology
Electroencephalography	To verify electrical inactivity or electrical silence in the brain, according to the technical standards of the Brazilian Society of Clinical Neurophysiology
Transcranial Doppler ultrasound	To verify the absence of intracranial blood flow due to the presence of reverberant diastolic flow, or small peaks in the initial phase of systole, as established by the Scientific Department of Neurosonology of the Brazilian Academy of Neurology
Brain scintigraphy/SPECT	To demonstrate the absence of brain perfusion or metabolism, according to the technical standards of the Brazilian Society of Nuclear Medicine

* In Brazil, the diagnosis of brain death is based on the presence of coma, absence of brainstem function, and absence of respiratory movements on an apnea test in two different clinical examinations. The performance of at least one ancillary test to unequivocally demonstrate the absence of blood perfusion or electrical or metabolic brain activity is mandatory.

Despite the regional variability, brain death protocols are often initiated only after imaging of the brain by CT or MRI has clearly demonstrated devastating cerebral injury consistent with cerebral circulatory arrest^(1)^. In addition, the diagnosis of brain death is important for organ donation^(7)^. Therefore, this article reviews the key neuroimaging features of brain death, with an emphasis on CT and MRI findings.

## PATHOPHYSIOLOGY OF BRAIN DEATH

Brain death can occur due to a primary insult—e.g., subarachnoid hemorrhage, traumatic brain injury, intracerebral hemorrhage, and ischemic stroke—or due to a secondary insult—e.g., cardiac arrest and global brain anoxia^(1)^. Regardless of the cause, the final common pathway of brain death involves a large increase in intracranial pressure, which impedes cerebral circulation and reduces cerebral perfusion pressure, resulting in secondary anoxic brain injury when the intracranial pressure exceeds the mean arterial pressure and cerebral perfusion stops^(8)^.

The pathophysiology of brain death is related primarily to a combination of hypoxia and ischemia in the setting of extensive brain injury, as well as to the effects of longstanding brain edema, which increases intracranial pressure, regardless of its cause^(2,9)^. In response to the increased intracranial pressure, the mean arterial pressure rises in an effort to maintain cerebral perfusion pressure^(2)^. However, because the adult brain is confined to a rigid structure (the skull), there is limited ability to compensate for the increasing brain volume as edema progresses^(10)^. When the main compensatory processes for the maintenance of constant intracranial pressure, such as cerebrospinal fluid reabsorption, fail, the cerebral perfusion pressure decreases. Eventually, a threshold is reached; small increases in brain volume lead to exponential increases in intracranial pressure, and brain herniation occurs^(11)^. When the cerebral perfusion pressure becomes insufficient, brain ischemia occurs, usually in a rostrocaudal direction^(2,12)^.

Global brain injury leads to marked swelling and the destruction of neurons, with the irreversible loss of physiological brain activity, resulting in electrocerebral silence, despite the continuation of advanced life support. Brainstem damage causes respiratory and cardiac center disruption, together with injury to the reticular activating system, leading to the loss of brainstem reflexes^(2,12)^.

## PATHOLOGY OF BRAIN DEATH

**Figure 1:**
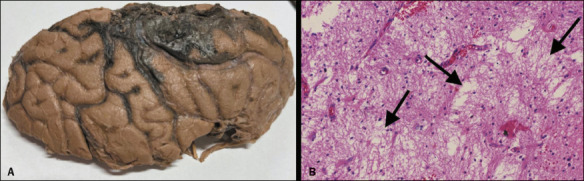
A: Gross brain pathology of the right brain hemisphere of a patient diagnosed with brain death and submitted to necropsy, demonstrated widened gyri with fl attened surfaces and narrowed sulci, characteristic of edema, accompanied by hemorrhage. B: Histopathology of the cerebral white matter in other patient diagnosed with brain death and submitted to necropsy demonstrated marked areas of edema (arrows; hematoxylin and eosin staining; magnifi cation, ×100). These pathological features are nonspecifi c and are not pathognomonic of brain death.

Brain death has no distinctive pathological feature and cannot be diagnosed by neuropathological examination^(2)^. Macroscopically, postmortem examination reveals uncal and tonsillar herniation, which lead to brainstem compression, together with stretching and tearing of the pontine perforating branches of the basilar artery, resulting in pontine Duret hemorrhage^(2,13)^. In brain death, the brain is congested, soft, and friable. Microscopically, brain death presents as ischemic changes characterized by neuron shrinkage, with chromatin aggregation and cytoplasmic eosinophilia, as well as interstitial edema^(14)^, as illustrated in Figure 1.

In addition to the nonspecificity of the features of brain death, the pathological features of this condition are not distributed uniformly. However, lesions have been found to be more severe in the frontal, temporal, parietal, and occipital lobes, as well as in the basal ganglia, than in the thalami, midbrain, pons, medulla oblongata, and cerebellum^(14)^.

## THE ROLE OF NEUROIMAGING IN BRAIN DEATH

Due to the regional variation in brain death criteria and the ancillary tests approved for its determination, the use of neuroimaging examinations in this context is controversial. In many countries, it is recommended only in specifi c circumstances, whereas in others it is legally required for the diagnosis^(15)^. In most European countries, although the diagnosis of brain death is based on clinical features, neuroimaging has been used prior to defi nitive clinical examination, as a prerequisite to determine whether the imaging alterations are consistent with brain death or the cause of coma^(16)^. In the United States, the occurrence of catastrophic brain injury of known cause, consistent with cerebral circulatory arrest, must be identifi ed by standard neuroimaging prior to brain death assessment, and clinical determination or ancillary testing may be considered only after CT or MRI has been performed and has clearly demonstrated a devastating cerebral insult^(1)^. In Brazil, the diagnosis of the lesion responsible for coma must be established by clinical evaluation and confi rmed by neuroimaging or other diagnostic methods^(17)^. Standard neuroimaging is of great value in particular clinical scenarios, such as when the neurological evaluation is diffi cult or cannot be performed (e.g., because of multiple facial fractures), when intoxication of any origin cannot be excluded as the cause of coma, when cervical spinal cord injury has occurred or is suspected, and when the clinical picture leads to clinical uncertainty, due to spontaneous or refl ex movements^(15)^. Therefore, CT and MRI features associated with brain death can be important for identifying catastrophic brain injury consistent with cerebral circulatory arrest. However, imaging fi ndings cannot be used to diagnose brain death in the absence of clinical evidence.

## CT

Unenhanced brain CT can provide evidence of irreversible brain damage and of the primary intracranial event that caused brain death^(15)^. Because CT is widely available, it is commonly used for the initial examination of comatose patients^(9)^. When brain death is suspected, the initial interpretation of unenhanced brain CT examinations is essential for confi rming the occurrence of a catastrophic brain event and determining its underlying cause^(18)^.

**Figure 2:**
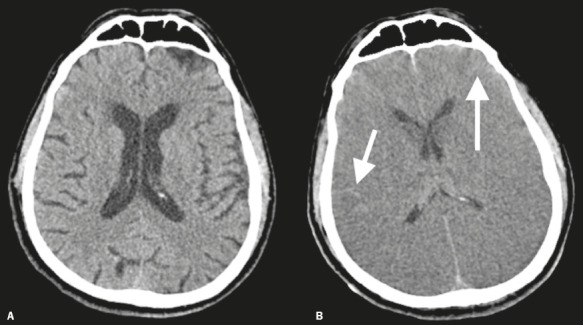
Brain CT of a 75-year-old man with COVID-19 and severe acute respiratory syndrome. A: The scan performed at the time of hospital admission was unremarkable, except for the demonstration of age-related changes. B: The patient was admitted to the intensive care unit and did not regain consciousness despite the cessation of sedation. A second brain CT demonstrated diffuse cerebral edema, swollen gyri, effaced sulci, compressed ventricles, and pseudosubarachnoid hemorrhage due to venous congestion (arrows). Thereafter, brain death was clinically diagnosed. Although brain CT is not an ancillary test for brain death determination, the neuroimaging-based explanation of coma is a prerequisite to brain death evaluation.

In most cases, CT provides evidence of the primary brain insult, such as single or multiple hemispheric lesions (e.g., intracerebral hemorrhage, stroke, tumor, and edema). It can also provide evidence of primary or secondary global hypoxic–ischemic injury, such as diffuse cerebral edema; loss of the gray–white matter boundary; effacement of the sulci, ventricles, and basal cisterns; and the “reversal” or “white cerebellum” sign. Pseudosubarachnoid hemorrhage can be seen secondary to hyperdense veins in effaced sulci. In addition, brain herniation, such as transtentorial herniation and herniation through the foramen magnum, occurs secondary to increasing intracranial pressure^(9,19)^, as can be seen in Figure 2. A CT perfusion scan enables functional assessment of the brain; although it demonstrates the absence of brain perfusion and confi rms severe hemodynamic arrest, its use in this context needs to be validated in larger-scale studies^(2,20)^. In addition, not all CT scanners employed in clinical practice are capable of performing perfusion studies and CTA is generally preferred.

The American Academy of Neurology has included neuroimaging-based explanation of coma as a prerequisite to brain death evaluation^(4)^. However, the clinical assessment of brain death remains of utmost importance^(2)^. The interpretation of CT fi ndings requires careful consideration of confounding factors, because unenhanced CT is of limited use beyond the radiological evaluation of inciting causes, such as brain edema, trauma, and ischemia.

Relatively normal brain CT findings should cast doubt on a brain death diagnosis, and an alternative diagnosis should be considered. However, brain CT may be falsely normal in certain circumstances, such as after cardiopulmonary arrest and acute stroke^(2,21)^.

## CTA

**Figure 3:**
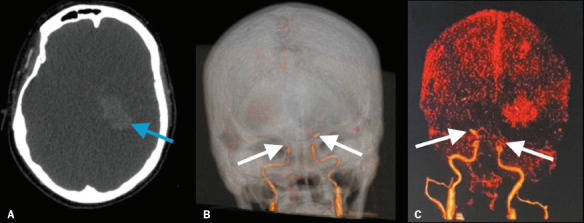
Brain CTA of a 37-year-old woman with deep vein thrombosis in the left leg, complicated by pulmonary thromboembolism. The patient was intubated and did not regain consciousness after the cessation of sedation. A: Brain CT examination showing diffuse brain edema, effaced sulci, compressed ventricles, and a left basal ganglia hematoma (arrow). B , C: Three-dimensional volume-rendered brain CTA demonstrating the absence of intracranial vessel enhancement above the supraclinoid segment of the internal carotid arteries (arrows). There were no brainstem refl exes, an electroencephalogram was isoelectric, and brain death was confi rmed clinically.

Having the advantages of being widely available, minimally invasive, rapid, and relatively easy to perform, even in critically ill patients, CTA can be performed in conjunction with brain CT in cases of suspected brain death. Several authors have proposed criteria for the diagnosis of brain death using CTA^(22,23,24)^. Those criteria are based on the absence of opacifi cation in the vertebrobasilar arteries, within the dura mater, and in the internal carotid arteries, above the supraclinoid segments, as well as in the deep veins^(3,9)^, as shown in Figure 3. However, the vessels to be considered vary among studies.

Dupas et al.^(22)^ used CTA in two phases (20 s and 54– 60 s after initial contrast injection) to diagnose brain death, using a score based on the lack of opacifi cation in seven intracerebral vessels—the two pericallosal arteries, the cortical segments of the two middle cerebral arteries, the two internal cerebral veins, and the great cerebral vein—which indicates stagnation and arrest of the contrast medium at the level of the internal carotid and vertebral arteries, as well as the absence of venous blood return. The authors demonstrated that this technique has 100% specifi city for the diagnosis of brain death^(22)^. Frampas et al.^(23)^ created a simplifi ed 4-point scale based on the lack of opacifi cation of the internal cerebral veins and of the cortical segments of the middle cerebral arteries. Other authors proposed a 10-point scale based on assessment of the postcommunicating segments of anterior cerebral arteries, the cortical segments of the middle cerebral arteries, the ambient segments of the posterior cerebral arteries, the basilar artery, the internal cerebral veins, and the great cerebral vein^(24)^. The 4-point scale has been found to be more sensitive than are 7- and 10-point scales, with a sensitivity of 96.3% versus 74.4% and 67.1%, respectively^(25,26)^. However, the use of the 4-point scale does not involve evaluation of the posterior circulation and therefore does not enable the consideration of whole-brain death^(9)^.

The variations in CTA performance and interpretation lead to great variation in the sensitivity of this modality for the diagnosis of brain death. Despite its low sensitivity, CTA can be useful in the diagnosis of brain death. Therefore, some countries, such as the Netherlands, France, Switzerland, and Canada, have approved its use as an ancillary test^(2)^. Other countries, such as Brazil and the United States, have not approved CTA as an ancillary test for the diagnosis of brain death, mainly because of insuffi - cient diagnostic confi dence^(6)^, given the lack of consensus on the radiographic criteria used in order to demonstrate the absence of intracranial blood fl ow. Nevertheless, CTA has some disadvantages, such as the fi lling of the major arteries near the skull base, mainly following decompressive craniectomy, even when the clinical criteria for brain death are met, and its potential for producing false-positive results in patients with hypotension^(3)^.

## MRI

The brain MRI features of brain death are parallel to the CT fi ndings, demonstrating the primary insult that led to brain death or secondary hypoxic–ischemic injury^(9)^. However, MRI has some disadvantages relative to CT^(2)^: it is more time consuming; it is not as widely available; and it is diffi cult to perform in critically ill patients and patients on mechanical ventilation.

An MRI scan can also show poor gray/white matter differentiation, sulci and ventricle effacement, and brain herniation^(27,28)^. A T2-weighted MRI sequence can demonstrate the absence of intracranial vascular fl ow voids in the major arteries in the basal cisterns, indicative of the absence of cerebral blood fl ow. Restricted diffusion secondary to cytotoxic edema can also be seen in the white matter, gray matter, brainstem, and cerebellum^(2,9,29)^. Susceptibility-weighted imaging has been described as an important tool for the acquisition of specifi c fi ndings related to brain death that should be included in protocols for the assessment of patients in this clinical scenario. It can show prominent hypointense signals in the medullary veins, parallel or perpendicular to the outer walls of both lateral ventricles, due to increased oxygen extraction, venous stasis, or venous dilatation secondary to the release of substances (e.g., adenosine) after neuronal death^(30)^.

**Figure 4:**
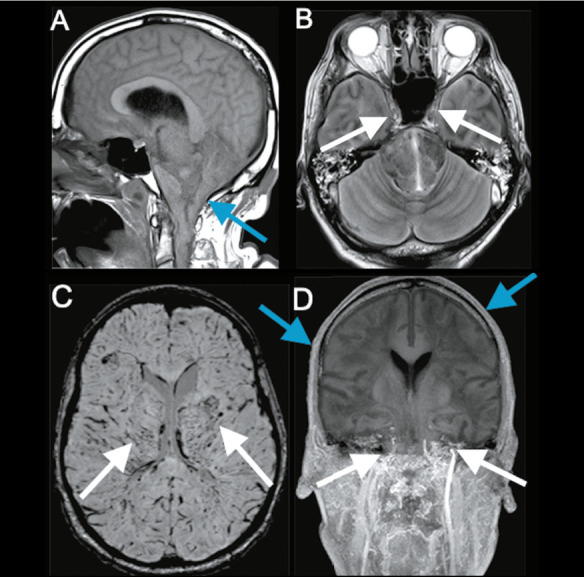
Brain MRI of a 71-year-old man who had a 15-min cardiac arrest caused by pulmonary sepsis. After two weeks, despite a reduction in the level of sedation, the patient did not regain consciousness. A: T1-weighted image demonstrating tonsillar herniation through the foramen magnum (arrow). B: T2-weighted image showing the loss of intra-arterial fl ow-voids in the internal carotid arteries (arrows) and cortical sulci effacement. C: Susceptibility-weighted imaging showing prominent hypointense signals in the deep medullary veins (arrows). D: Contrast-enhanced T1-weighted image demonstrating the absence of intracranial vessel enhancement. Note that the gadolinium-based contrast did not advance past the extracranial portions of the internal carotid arteries (white arrows) and that there is intense enhancement around the scalp (blue arrows). Brain death was confi rmed clinically.

After intravenous injection of gadolinium-based contrast, brain MRI can demonstrate intense enhancement around the nose and scalp—a parallel to the “hot nose” sign seen in scintigraphy^(29)^—and extracranial carotid artery enhancement with the absence of intracranial contrast enhancement^(2,29)^, as illustrated in Figure 4. Perfusion MRI may also show the absence of intracranial perfusion in the supratentorial and infratentorial compartments^(2)^. Although most MRI scanners can be used to perform perfusion studies, the utility of these studies in brain death evaluation needs to be validated in larger studies. It should be borne in mind that none of those MRI signs is pathognomonic of brain death, given that they can be seen in other scenarios, such as severe hypoxic–ischemic brain damage^(2)^.

## MRA

There are two ways in which to perform MRA^(31)^: after intravenous injection of gadolinium-based contrast; and using time-of-fl ight algorithms without contrast injection. In either case, MRA has the disadvantages of being diffi cult to perform in critically ill patients, having long scan times, and not being widely available, as well as being subject to susceptibility artifacts and the fact that varying criteria are used in order to document intracranial circulatory arrest^(2,9)^.

**Figure 5:**
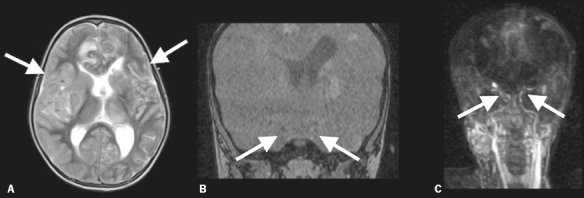
Brain MRI and MRA of a 10-year-old boy with bacterial meningitis. Despite the cessation of sedation, the patient did not regain consciousness. A: T2- weighted MRI showing extensive cortical brain edema and effacement of cortical sulci (arrows). B: Time-of-fl ight MRA demonstrated the absence of intracranial vessels fl ow (arrows). C: Four-dimensional MRA showed that the gadolinium-based contrast did not enter the intracranial portions of the internal carotid arteries (arrows). There were no brainstem refl exes, an apnea test was positive, and an electroencephalogram was isoelectric.

An MRA examination can demonstrate the absence of fl ow and/or enhancement in the intracranial arteries, although, as with other forms of angiography, some fi lling of proximal intracranial vessels may occur near the skull base^(9,31)^. Time-of-fl ight MRA does not show the intracranial vessels above the level of the supraclinoid segment of the internal carotid arteries^(32,33)^, and gadolinium-enhanced MRA does not show intracranial enhancement above the level of the anterior cerebral arteries and proximal segments of the middle cerebral arteries^(34)^, as shown in Figure 5. Magnetic resonance venography does not show the opacifi cation of the dural sinuses and does not enable visualization of the intracranial veins^(33)^.

Similar to CTA, MRA is associated with insuffi cient diagnostic confi dence to confi rm brain death. Therefore, it is recommended that radiologists refer strictly to brain blood-fl ow test results and avoid using the terminology “consistent with brain death” when referencing the cerebral blood fl ow^(3)^.

## NEUROIMAGING AS AN ANCILLARY TEST FOR BRAIN DEATH

In some countries, such as the United States, ancillary tests are used in the determination of brain death when uncertainty exists about the reliability of neurological examination or when an apnea test cannot be performed. Ancillary tests cannot replace neurological examination, and the possibility of false-positive results must be considered. According to the American Academy of Neurology, the time of death is defi ned as the time at which the arterial partial pressure of carbon dioxide reaches the target value on an apnea test, or when the ancillary test results have been offi cially interpreted^(4)^. In Brazil, the use of at least one ancillary test is mandatory, and the time of death is defi ned as the time at which the last procedure was performed during the determination of brain death. The ancillary test is chosen on the basis of the clinical situation and local availability^(17)^.

## DSA

Although DSA is usually considered to be the gold standard for the evaluation of intracranial fl ow, it has the disadvantages of being invasive, being time consuming, and requiring specifi c expertise, as well as carrying a risk of contrast-induced renal injury in potential organ donors^(2,9)^. For brain death assessment with DSA, the contrast medium should be injected into the aortic arch under pressure and should reach the anterior and posterior circulations. The results are considered positive when there is no intracerebral contrast fi lling, including the arterial fl ow at the level of entry of the carotid and vertebral arteries into the skull, and no venous drainage^(4,35)^, due to increased intracranial pressure and the destruction of the intracerebral vessels, in conjunction with necrosis. The external carotid circulation can be patent^(2)^. Like CTA and MRA, DSA can yield false-positive results in patients with hypotension and false-negative results in patients who have undergone decompressive craniectomy^(36)^. In addition, some proximal opacifi cation of the intracranial arteries due to stasis fi lling can be seen on DSA in brain dead patients^(2,9,35)^.

### Transcranial Doppler ultrasound

Transcranial Doppler ultrasound can be performed through the temporal windows, above the zygomatic arch, to assess the middle cerebral arteries, and through the suboccipital window to assess the vertebrobasilar arteries^(31)^. It has the advantages of being rapid, easily repeated if necessary, and performed at the bedside, which is important for use in physiologically unstable patients. However, this method is operator dependent, requires specifi c training, and is not available at all hospitals; in addition, some patients do not have good ultrasound windows^(9)^.

In brain death, transcranial Doppler imaging can demonstrate reverberant fl ow, also called the “to-and-fro” pattern, characterized by a spectrum of two-phase fl ow velocities, with equivalent components of inward and outward fl ow and a zero average velocity; short (< 100 cm/s) spikes at the beginning of the systolic phase with no fl ow in the remaining cardiac cycle; or the disappearance of a previously detected fl ow^(37)^. However, an isolated transcranial Doppler ultrasound examination demonstrating a lack of fl ow should not be considered to be indicative of brain death, given that some patients have no suitable bone window^(31)^.

### Brain scintigraphy/SPECT

Nuclear medicine tests have the advantage of measuring both brain metabolism and blood fl ow. However, scintigraphy and SPECT have some disadvantages, such as logistics problems, their time-consuming nature, and the need to use radiopharmaceuticals that can cross the blood–brain barrier, such as technetium-99m hexamethyl propylene amine oxime and technetium-99m ethyl cysteinate dimer, which may create issues at some facilities, because these radiopharmaceuticals may not be readily available^(2,15)^. In brain death, scintigraphy and SPECT typically demonstrate no uptake in the brain and cerebellum (the “hollow-skull” sign). The “hot nose” sign, described as increased uptake in the nasal area, with no uptake in the intracranial arteries, is also a feature of brain death^(3,38)^. However, the “hot nose” sign plays a limited role in the determination of brain death, because it may occur as evidence of any cause of increased intracranial pressure, such as ischemic stroke, subdural hematoma, and hepatic encephalopathy^(39)^. Scintigraphy and SPECT also have equivocal patterns of radiopharmaceutical uptake, such as the preservation of cerebellar perfusion without cerebral perfusion and the absence of cerebellar perfusion with the preservation of cerebral uptake^(40)^.

## CONCLUSION

Although the final diagnosis of brain death depends on clinical findings, radiologists may play an important role in the initial recognition of global hypoxic–ischemic injury and of the absence of cerebral perfusion. In Brazil, where at least one ancillary test must be performed for the diagnosis of brain death, the approved ancillary tests are cerebral DSA, transcranial Doppler ultrasound, brain SPECT, and electroencephalography. However, CTA is widely available, is commonly used in comatose patients, and has been recognized as an ancillary test for the determination of brain death in other countries.
